# General lack of global dosage compensation in ZZ/ZW systems? Broadening the perspective with RNA-seq

**DOI:** 10.1186/1471-2164-12-91

**Published:** 2011-02-01

**Authors:** Jochen BW Wolf, Jarosław Bryk

**Affiliations:** 1 Max Planck Institute for Evolutionary Biology, Department of Evolutionary Genetics, August-Thienemannstr. 2, 24306 Plön, Germany; 2 Evolutionary Biology Centre, Department of Evolutionary Biology, Uppsala University, Norbyvägen 18 D, 75236 Uppsala, Sweden

## Abstract

**Background:**

Species with heteromorphic sex chromosomes face the challenge of large-scale imbalance in gene dose. Microarray-based studies in several independent male heterogametic XX/XY systems suggest that dosage compensation mechanisms are in place to mitigate the detrimental effects of gene dose differences. However, recent genomic research on female heterogametic ZZ/ZW systems has generated surprising results. In two bird species and one lepidopteran no evidence for a global dosage compensating mechanism has been found. The recent advent of massively parallel RNA sequencing now opens up the possibility to gauge the generality of this observation with a broader phylogenetic sampling. It further allows assessing the validity of microarray-based inference on dosage compensation with a novel technology.

**Results:**

We here expemplify this approach using massively parallel sequencing on barcoded individuals of a bird species, the European crow (*Corvus corone*), where previously no genetic resources were available. Testing for Z-linkage with quantitative PCR (qPCR,) we first establish that orthology with distantly related species (chicken, zebra finch) can be used as a good predictor for chromosomal affiliation of a gene. We then use a digital measure of gene expression (RNA-seq) on brain transcriptome and confirm a global lack of dosage compensation on the Z chromosome. RNA-seq estimates of male-to-female (m:f) expression difference on the Z compare well to previous microarray-based estimates in birds and lepidopterans. The data further lends support that an up-regulation of female Z-linked genes conveys partial compensation and suggest a relationship between sex-bias and absolute expression level of a gene. Correlation of sex-biased gene expression on the Z chromosome across all three bird species further suggests that the degree of compensation has been partly conserved across 100 million years of avian evolution.

**Conclusions:**

This work demonstrates that the study of dosage compensation has become amenable to species where previously no genetic resources were available. Massively parallele transcriptome sequencing allows re-assessing the degree of dosage compensation with a novel tool in well-studies species and, in addition, gain valuable insights into the generality of mechanisms across independent taxonomic group for both the XX/XY and ZZ/ZW system.

## Background

Sex determining mechanisms have evolved many times independently and range from environmental or social clues to genetic hard-wiring [1,2]. The two most common forms of chromosomal sex determination are the male heterogametic XX/XY system and the ZZ/ZW system, where females are the heterogametic sex. A widely accepted model posits that in the course of evolution an autosomal chromosome acquired a sex determining locus and was henceforth doomed to degrade by mutational silencing and deletions to a point where it now only harbors a handful of essential genes [3]. This results in a large-scale sexual imbalance in gene dose, which, if not countered, should entail severe detrimental effects [4-6]. Compensating mechanisms are thus expected to be in place that reduce the disparity in sex-linked gene expression and thereby mitigate the effect of sexual imbalance in gene dose. It thus did not come as a surprise that diverse epigenetic dosage compensation mechanisms have been discovered in several XX/XY systems as dissimilar as *Drosophila*, *Mus *and *Caenorhabditis *and much progress has been made to understand their evolutionary basis [7].

The recent embarquement on genome wide-research on female heterogametic ZZ/ZW systems, however, has generated unexpected results that reinstated much interest in the phenomenon [8-10]: two bird species, chicken and zebra finch, seem to entirely lack a global dosage mechanism [11,12]. Though varying across life stages and tissues [13,14], m:f ratios of Z-linked gene expression consistently deviate from unity. Microarray studies as well as quantitative PCR (qPCR) based studies suggest values somewhere in between 1 (full compensation) and 2 (absence of compensation). This indicates either a lower efficiency in dosage compensation, or that the buffering capacity of gene interaction networks alone can compensate for dosage differences [15]. Effective regional dosage compensation has been described around the male hyper-methylated (MHM) locus in chicken [16], but no such regional patterns are found in zebra finch [14].

Independent evidence for a lack of wholesale dosage compensation comes from the Lepidopteran *Bombyx mori *[17] and has prompted the idea that species with ZZ/ZW sex chromosomes are fundamentally different from male heterogametic XX/XY systems in the way they deal with differences in gene dosage [10]. At present, such an inference may, however, be premature, as phylogenetically independent sample size is low (two bird species, one moth). It thus remains an open question, if ZZ/ZW systems in general have evolved similar chromatin-modifying mechanism as in XX/XY systems [18], or if the general buffering capacity of protein interaction networks described for unbalanced dosage of autosomal genes in partial aneuploids [19,20] sufficiently counteracts the detrimental effects of dosage differences in the heterogametic sex. Broader taxonomic sampling is needed to understand the diverse mechanisms by which ZZ/ZW species deal with gene dosage imbalances, but also to test for the generality of effective compensation mechanisms in further XX/XY systems [21].

The rapid advances in sequencing technology over the last few years open up this possibility and allow validating microarray-based inference with a novel methodological tool. Xiong et al. 2010 [22] recently applied RNA-seq data in the context of dosage compensation. Using datasets on well established models such as worm and mouse, they reach the conclusion that apparent compensation of gene dosage differences between the X chromosome and autosomes may simply reflect an artifact of the microarray technique. RNA-seq data suggested no dosage compensation of the active X chromosome. This striking result clearly demonstrates that the addition of a novel technology to the study of dosage compensation will prove valuable and has the potential to even overthrow current belief.

RNA-seq further allows addressing dosage compensation in a much broader set of taxa on a genome-wide scale [reviewed in [23]]. In contrast to microarrays which are traditionally restricted to models organism with rich genomic resources, massively parallel transcriptome sequencing yields sequence information and concomitantly provides a digital measure of gene expression for basically any species [24]. In principle, this allows inferring gene identity by using orthology relationships to related species with annotated genomes and at the same time determine expression levels.

We here demonstrate the applicability of such an approach for the study of dosage compensation in a non-model bird species, the European Crow (*Corvus corone*). Using RNA-seq data from massively parallel pyrosequencing we examine patterns of autosomal and Z-linked gene expression in brain and address how RNA-seq based estimators of dosage compensation compare to values obtained from microarray or qPCR experiments. We discuss the findings with special reference to the results from the two other bird species and highlight the potential and the limitations of this approach.

## Results and discussion

### Conserved synteny

Karyotypes and synteny have been surprisingly well conserved during more than 100 million years of avian evolution [25]. This allows using orthology information from phylogenetically distant bird species with annotated genomes to assess with high confidence on which chromosome a gene is located. As knowledge on chromosomal affiliation is pivotal to the study of dosage compensation, we explicitly tested this assumption. We took a subset of 21 putatively Z-linked genes as inferred by 1:1 orthology with chicken and zebra finch and verified Z-linkage by comparing the copy number of a gene relative to an autosomal control with qPCR. Our prediction was that the ratio of copy number of any Z-linked gene to an autosomal gene should be equal to 0.5 in females (ZW) and to 1 in males (ZZ). Since differences in amplification efficiency between the Z-linked locus and the autosomal control may lead to slight deviations from theses values, the more decisive measure is the male-to-female ratio (m:f) which is 1 for autosomal loci and 2 for Z-linked loci (E [ZZ_conc_/ZW_conc_.] = 2). Of the 21 genes tested, 20 showed the ratio expected for a Z-linked locus and one gene (ENSTGUG00000017495) was inferred to be autosomal (Table 1). This gene is homologous to asparaginyl-tRNA synthetase, which is annotated as Z-linked in both zebra finch and chicken. Several lines of evidence suggest that autosomal linkage in crows is real and not simply owing to a methodological artifact: the gene has no annotated orthologue on the W chromosome neither in chicken nor in zebra finch, all six independent primer combinations yielded consistent results (Table 1) and sequence data for the three longest PCR amplification products all correctly mapped to the Z-linked 1:1 orthologue in zebra finch and chicken. We also PCR-amplified seven exon-exon junctions of ENSTGUG00000017495, and in all cases obtained products lengths consistent with the presence of introns and of similar size as expected from intron length in zebra finch (R^2 ^= 0.93, p < 0.001). This suggests that this gene did not undergo retrotransposition, but may instead have entirely moved from an ancestral Z chromosomal state to an autosomal location in the lineage leading to crow.

**Table 1:** Copy number variation for 21 putatively Z-linked genes as inferred by orthology with zebra finch.

gene	locus	Mean m:f	f	m	m:f	t	p-value
ENSTGUG00000000043	exon3	2	0.47	0.95	2.03	5.48	0.00077
	exon4		0.70	1.69	2.40	4.14	0.00305
ENSTGUG00000000468	exon4	2	0.45	0.80	1.79	5.37	0.00085
	3'UTR		0.28	0.57	2.02	6.60	0.00029

ENSTGUG00000000630	exon4	2	0.48	0.96	1.97	10.99	0.00002

ENSTGUG00000000903	3'UTR	2	0.22	0.46	2.10	4.51	0.00203
	exon26		0.44	0.86	1.94	5.49	0.00077

ENSTGUG00000001006	exon4	2	0.31	0.59	1.92	6.62	0.00029
	3'UTR		0.50	1.00	2.03	17.43	0.00000

ENSTGUG00000001006	exon1	2	0.30	0.66	2.20	5.58	0.00071

ENSTGUG00000001019	exon4	2	0.34	0.64	1.90	8.37	0.00008
	exon5		0.48	0.96	1.98	8.73	0.00006

ENSTGUG00000001401	3'UTR	2	0.50	1.00	1.99	4.71	0.00164
	3'UTR		0.23	0.48	2.09	10.07	0.00003

ENSTGUG00000001582	exon5	2	0.30	0.66	2.20	9.70	0.00003

ENSTGUG00000001587	3'UTR	2	0.26	0.56	2.17	5.66	0.00065

ENSTGUG00000001918	3'UTR		0.40	0.78	1.95	5.34	0.00088
	3'UTR	2	0.39	0.79	2.04	7.83	0.00011
	3'UTR		0.49	1.03	2.11	6.62	0.00029

ENSTGUG00000002186	exon2	2	0.26	0.64	2.45	30.20	0.00000
	exon2		0.29	0.67	2.32	8.24	0.00009

ENSTGUG00000002229	3'UTR	2	0.48	1.00	2.09	4.55	0.00195
	exon5		0.33	0.69	2.07	4.04	0.00339

ENSTGUG00000003373	exon5	2	0.38	0.77	2.05	13.08	0.00001

ENSTGUG00000003968	exon3	2	0.42	0.93	2.21	7.34	0.00016

ENSTGUG00000004334	exon4	2	0.42	0.92	2.18	7.60	0.00014

ENSTGUG00000006083	exon5	2	0.39	0.84	2.15	9.66	0.00004

ENSTGUG00000006814	exon2	2	0.44	0.90	2.03	4.82	0.00148

ENSTGUG00000006923	exon4	2	0.51	1.05	2.06	11.07	0.00002

	exon12		0.73	0.83	1.14	1.17	0.14404
	exon13		1.51	1.60	1.06	0.65	0.26995
	exon17	1	1.75	1.85	1.05	0.41	0.34855
ENSTGUG00000017495	3'UTR		2.21	2.35	1.07	0.53	0.30709
	3'UTR		0.68	0.76	1.12	0.72	0.24832
	exon9		1.70	1.80	1.06	0.50	0.31822

ENSTGUG00000017541	3'UTR	2	0.40	0.88	2.21	6.98	0.00021

*gene: *Z-linked gene in zebra finch for which orthology was inferred with crow: l*ocus: *specific location of the primers used for qPCR as inferred from orthology with zebra finch; *mean m:f: *male-to-female ratio in copy number averaged over all primer pairs tested for a given gene; *f, m: *average copy number relative to an autosomal control for a sample of four females and four males, respectively. Under the assumption of equal amplification efficiency the expected values are 0.5 for females (ZW/AA) and 1 for males (ZZ/AA). *m:f: *male-to-female ratio in copy number (expected to be 2 for Z-linked genes). *t, p-value*: t-statistic and type I error (6 d.f.) for the comparison of relative copy numbers between males (*m*) and females (*f*). Primer sequences for all loci can be found in Additional file 1 (Table 1).

In birds, movement of complete genes (including introns) between sex chromosomes and autosomes seems to be rare, but it does happen. During more than 100 mya of evolution between chicken and zebra finch approx. 2% (15/745) of 1:1 orthologues have moved between the Z chromosome and autosomes (BioMart ENSEMBL Genes 57). Assuming half the divergence time between crows and zebra finch, one would expect syntenic disruption for ~1% of Z-linked genes. Under this premise, finding one aberrant gene in a set of 21 is not unexpected (p = 0.37, Fisher exact test).

Concluding, orthology relationships between zebra finch and chicken are on the whole a good indicator of chromosomal affiliation of a gene in crows and allow for analyses of gene expression on a chromosome-by-chromosome basis. This opens the door to dosage compensation analyses in this species where an annotated genome is not available. Nonetheless, one should be aware that synteny is not perfect, and Z-linkage may be wrongly inferred for a small number of genes. While this may slightly influence the m:f expression ratio on the Z, it will not compromise the general validity of sex-specific expression analyses.

### Dosage compensation

Compared to gonads and other somatic tissue, brain is less susceptible to sex-specific expression patterns [11] and should thus be better suited to concentrate on effects of dosage differences between the sexes rather than on sex bias in expression owing to sexual selection as e.g. in gonads (but see [26] describing the weak relationship between sexual antagonism and sex bias in gene expression). We therefore used brain transcriptome data to examine genome-wide patterns of sex bias in expression levels. From a total of 7564 transcripts for which orthology with chicken or zebra finch could be established, we obtained a digital measure of gene expression and inferred the gene's chromosomal location on the basis of orthologous relationships. Due to the high degree of conserved synteny (see above) the general results are unaffected of whether zebra finch or chicken are used as a reference. To reduce sampling variance for genes with very low sequencing coverage, a subset of 3556 moderately to highly expressed genes was used to explore the patterns of sex biased expression across autosomes and sex chromosomes.

#### Evidence for incomplete dosage compensation

**Figure 1:**
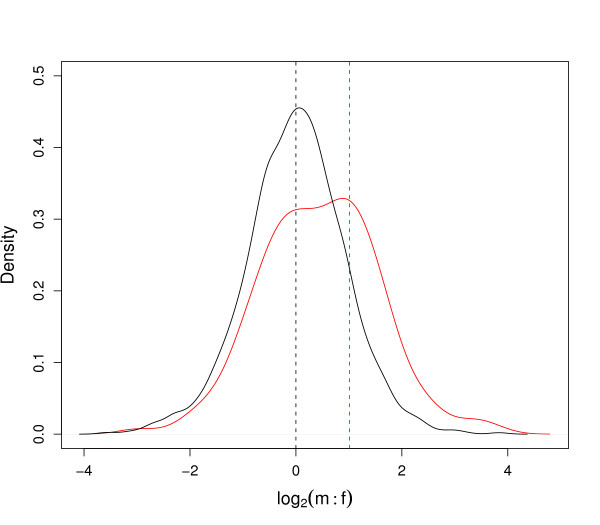
**Male-to-female ratio of gene expression for autosomal and Z-linked genes**. Best Gaussian kernel density function for log2(m:f) ratios of 3300 autosomal genes (black) and 156 Z-linked genes (red). A mean logarithmic male-to-female ratio (log2(m:f) of zero indicates that autosomal genes are equally expressed between females and males (black dotted line). Expression of Z-linked genes, on the contrary, is on average male biased (red dotted line) and indicates a lack of dosage compensation for most genes. The shape of the kernel is robust to different smoothing kernels and parameter settings.

**Figure 2:**
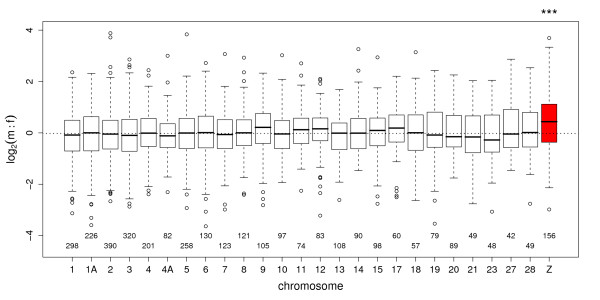
**Male-to-female ratio of gene expression by chromosome**. Male-to-female ratio (m:f) of gene expression in sub-adult crow brains grouped by orthologous chromosomal position in zebra finch. The m:f ratio is significantly elevated on the Z chromosome (***) while there is no significant deviation from expected equity in expression for any of the autosomes. Number of sampled genes per chromosome is indicated below (only chromosomes with more than 40 genes are shown). Boxes include the 2^nd ^and 3^rd ^quantile; whiskers extend to the most extreme data points that lie within 1.5 times the interquartile range from the box; points depict extreme values beyond that boundary.

For the 156 Z-linked genes, male expression was on average 1.36 times higher than female expression (95% CI: 1.20-1.55). This is significantly less than the expected two-fold difference for complete absence of dosage compensation, but differs significantly from 3400 autosomal genes with a mean m:f ratio of 1.00 (95% CI: 0.97-1.02). Slight bimodality in the distribution of m:f ratios of Z genes centering around 1 and 1.36 may indicate discrete differences in the degree of compensation for different classes of genes (Figure 1). Mean m:f estimates across different autosomes were similar (range: 0.89-1.10, Figure 2). Only the Z chromosome significantly deviated from expected unity (Figure 2; p < 0.001).

These results lend support to the hypothesis that the overall pattern of ineffective dosage compensation may indeed be a general pattern in birds. Our RNA-seq based estimates of m:f ratios compare rather well with values obtained from microarray studies in other bird species. Ellegren *et al*. 2007 [11] report a mean m:f ratio of 0.97-1.04 in autosomes and 1.42 for sex chromosomes in somatic tissue of chicken. Itoh et al. [12] report mean m:f ratios of 0.99/0.99 in autosomes and 1.40/1.23 in sex chromosomes of chicken and zebra finch, respectively. A microarray study on the Lepidopteran *Bombyx mori *suggests a mean m:f ratio of 1.41 for sex chromosomes of somatic tissue, which is very similar to the estimates in chicken. However, Itoh et al. [12] suggested that microarray data may underestimate the degree of sex-bias, as estimates of m:f ratios in half a dozen of Z-linked genes were considerably higher (~2) using qPCR. Our study adds information with a third independent method. The concordance of our RNA-seq based estimates with previous microarray-based data from other bird species is in contrast with findings from Xiong et al. 2010 [22] who report a striking difference between microarray and RNA-seq based estimates of X- to autosomal expression ratios. Being a digital measure of expression RNA-seq is accurate across a wider range of expression levels than in microarrays [27] and estimation variation seems to be considerably lower [22]. Still, it shall not be concealed that that potential statistical biases exist [e.g. [28]] and that accuracy depends on proper mapping and coverage, and that GC content and the gene set used (which in turn can be a function of coverage) influence expression ratios. More detailed studies contrasting expression patterns from microarray analyses and RNA-seq data are clearly needed in the context of dosage compensation to assess the relative bias associated with each method.

Regardless of which methodology is applied, the degree of dosage compensation suggested by m:f ratio values is below 1.5 for all avian species and one lepidopteran. This is similar to what has been observed in autosomal aneuploids and artificially induced imbalances of autosomal genes where dosage differences are also partially compensated [29]. This may suggest that intrinsic gene network resilience, auto-regulation through negative feedback and competition for limited regulatory factors [5] may sufficiently counter the imbalance in gene expression due to gene dosage differences in the heterogametic sex. Studies on the effects of autosomal gene dose, e.g. like those recently conducted in *Drosophila *[19,20], are highly important to evaluate the contribution of gene network resilience to mitigate potential dosage effects.

#### Upregulation of female expression

As seen above, m:f ratios of less than two indicate some degree of compensation either by passive resilience of gene interaction networks or by a specifically evolved mechanism. Lower than expected m:f ratios can either be achieved by up-regulation of female or by down-regulation of male Z-linked genes. The former scenario is more plausible though, as in the course of sex chromosome evolution, dosage imbalance in the heterogametic sex should have increasingly experienced counter-selection.

In males, log2 expression levels of Z-linked genes (Z) were lower in tendency, but not significantly different from those of autosomes (A) (mean ± 95% CI: Z_m _= 6.14 ± 0.24; A_m _= 6.22 ±, 0.06; Z_m_:A_m _= 0.95, 95% CI: 0.77-1.16)., In females, expression of Z-linked genes was significantly lower than of autosomal genes (mean ± 95% CI: Z_f _= 5.86 ± 0.24; A_f _= 6.21 ± 0.06.). A Z_f_:A_f _ratio of 0.78 (95% CI: 0.64-0.95) is significantly above the ratio given by the number of chromosomes and suggests up-regulation of Z-linked genes relative to autosomal genes in females.

Up-regulation of female Z-linked expression rather than a down-regulation of Z-linked genes in males has also been suggested for zebra finch (Z_m_:A_m _= 0.9-1.1; Z_f_:A_f _= 0.65-0.9) [14] and is theoretically predicted by a verbal model from Birchler et al. [30] that posits a predominant role of negative *trans*-acting effects. This model is based on the observation that in most cases *trans*-acting regulatory genes seem to act as repressors rather than activators and reduce the expression of target genes (e.g. *white *locus in Drosophila) [30]. Reduced dosage in such a *trans*-acting repressor gene would consequently lead to increased expression of the target gene. Translated into the context of sex-specific dosage compensation this would mean that *trans*-acting regulatory genes located on the Z should show on average increase the expression of their target genes in the heterogametic sex. In heterogametic females, where Z dosage is reduced compared to ZZ males, target genes are consequently expected to be up-regulated. If target genes are locally confined to the vicinity of the trans-acting gene on the Z chromosome, an up-regulation of Z specific genes in females rather than a down-regulation in males should indeed be observed. This fits the observation in zebra finch [14] and is consistent with our data. However, the assumption that target genes are confined to the vicinity of the *trans *acting gene within the same chromosome is arguably too restrictive. If target genes are similarly present on autosomes one would also expect up-regulation of autosomal target genes in heterogametic females. A male-to-female ratio in autosomal expression (A_m_:A_f_) of 1.00 (95% CI: 0.93-1.08) estimated from our data, similarly observed for zebra finch and chicken [14] does not indicate a large-scale up-regulation of autosomal genes in females.

#### Degree of compensation and level of expression

Previous studies have reported a relationship between the degree of sex bias and the level of expression, but the patterns are inconsistent between species. In zebra finch, male biased genes (m:f ≫ 1) have higher expression than unbiased genes in males, but lower expression in females [14,16]. A different pattern has been observed in silkworm where there was no difference between biased and unbiased genes in males, but lower expression for male-biased genes in females [17]. In chicken, Melamed & Arnold [16] find no difference between male-biased and unbiased genes for females, but higher expression levels for male-biased genes in males [16].

**Figure 3:**
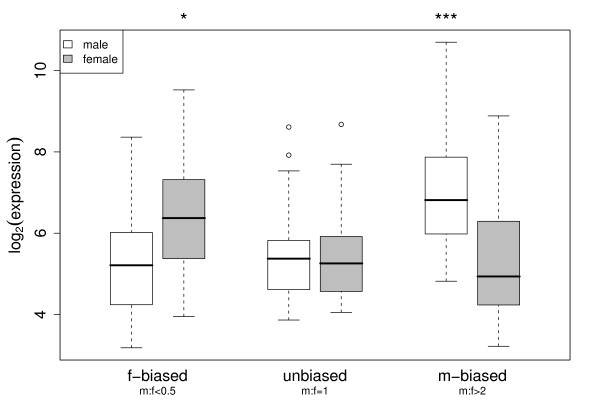
**Relationship between sex bias in gene expression and expression level**. Relationship of male-to-female ratios (m:f) with male (white) and female (gray) expression levels. While male expression is significantly higher for genes with high m:f ratio (m[ale]-biased) the opposite is indicated for genes expressed in females. Genes with a low m:f ratio (f[emale]-biased) are slightly more expressed in females. Asterisks indicate standard Type I error thresholds. Boxes include the 2^nd ^and 3^rd ^quantile; whiskers extend to the most extreme data points that lie within 1.5 times the interquartile range from the box; points depict extreme values beyond that boundary.

In male crows, male-biased genes (m:f > 2) are higher expressed than unbiased genes (0.8 < m:f < 1.2) (Figure 3; ANOVA: F_2,98 _= 18.6, R^2 ^= 0.27; Bernoulli post-hoc test: p < 0.001). In females, expression of male-biased and unbiased genes are indistinguishable, while expression of female-biased genes (m:f < 0.5) may be slightly reduced (ANOVA: F_2,98 _= 6.70, R^2 ^= 0.12; Bernoulli post-hoc test: p = 0.046). This pattern largely follows the observation of Melamed & Arnold [16]. Under a model where dosage compensated genes have on average a lower level of expression than non-compensated genes it would accordingly suggest that partial compensation may be initiated by females [16]. Such a scenario makes sense, since detrimental dosage effects are present only in the heterogametic sex and should entail a selective response in females.

However, the interpretation of the relationship between levels of gene expression and sex bias is not straightforward. High plasticity in expression levels depending on age and tissue and additional effects of sexual selection can create complex patterns. This - together with differences in statistical power - may be reflected in the discrepancy of observations between studies.

#### Conservation of m:f ratios across avian lineages

Itoh et al 2010 [14] compared m:f ratios across different tissues in chicken embryos and across different tissues and life-stages in zebra finch. Average correlation coefficients were considerably higher for Z linked genes (0.89 and 0.76 for chicken and zebra finch respectively) than for autosomal genes (0.32 and 0.08 respectively). This pattern may point towards a compensation mechanism that is robust across tissues and developmental stages. A similar conclusion can be drawn from cross-species comparisons. While no correlation between zebra finch and chicken could be found for autosomal genes, significant but weak correlations (mean r = 0.19) were found across tissues and life-stages between the two species.

We compared sex bias in gene expression for age - and tissue- matched samples of adult brain from zebra finch [31], chicken [13] and crows for Z-linked genes. We restricted the analysis to 41 unique Z genes that were present in crows and for which m:f ratios were available for ortholgous genes in zebra finch and chicken. Correlation coefficients ranged from 0.3 to 0.4 (Table 2) and were similar between crows and zebra finch when compared to the more distantly related chicken (r = 0.39, 0.40). The correlation of crow m:f ratios to the more closely related zebra finch was lower (r = 0.30). This is surprising at first sight, but confidence intervals for *r *largely overlap. Autosomal m:f ratios were available only for a 1265 orthologous between zebra finch and crow. Consistent with the comparison between chicken and zebra finch, the correlation was lower than for the Z chromosome (r = 0.11).

**Table 2:** Correlation of m:f ratios for 41 1:1:1 orthologues genes on the Z chromosome (Z) across zebra finch, chicken and crow and for 1265 autosomal (A) 1:1 orthologues between zebra finch and crow.

	Zebra finch	Chicken	Crow
**Zebra finch**	-	Z: 0.40	A:0.11
			Z: 0.30

**Chicken**	Z: p = 0.009	-	Z: 0.39

**Crow**	A: p < 0.001	Z: p = 0.001	-
	Z: p = 0.060		

Upper half: Pearson correlation coefficients for log2 (m:f); lower half: Type I error probabilities.

The fact that the correlation values for both the Z chromosome and autosomes are relatively high compared to previous studies (see above) may partly be explained by using tissue-and age-matched samples. It may, however, also reflect the conservative, rather highly expressed gene set that was selected by mapping success of crow contigs and 1:1 orthology between chicken and zebra finch. Pairwise comparison between chicken and zebra finch with a larger gene set of 109 genes, indicated a slightly lower correlation (r = 0.34) and could point towards the fascinating possibility that expression levels, gene sequence and dosage compensation may co-evolve.

Overall, cross-specific comparison of sex-biased gene expression on the Z chromosome suggests at least some degree of conservation across more than 100 million years of avian evolution and opens up an interesting avenue for comparative studies once m:f ratios are available for additional species.

#### Localized effects of dosage compensation

It has been suggested in chicken that dosage compensation in birds may be locally restricted to the evolutionary older stratum of the Z chromosome and that the distal end of the younger stratum harbors a concentration of non-compensated genes [16]. Such a mechanism, however, seems to be absent in zebra finch [14]. Under the assumptions that bird chromosomes are not only syntenic [32], but have undergone only minor intra-chromosomal rearrangements [25], we should in principle be able to study localized effects of dosage compensation also in non-model bird species. Extensive studies on linkage in two passerine species suggest that only minor rearrangements have occurred on the Z chromosomes between galliforms (chicken) and passerines (flycatcher: [33], great reed warbler [34], blue tit [35], Siberian Jay [36]), but two other passerine species where linkage information is available suggest large scale rearrangements on the Z [37,38]. Accordingly, patterns of m:f ratios differ strongly in crows depending on whether chromosomal gene locations are taken from orthologues in chicken or zebra finch (Figure 1 in Additional file 1). This discloses a clear limitation of the comparative approach using genetic non-model species. Even in avian taxa, where synteny is generally high [25], it will be difficult to pin down localized effects of dosage compensation unless detailed information on gene order is available. Therefore, species where multi-generational samples of related individuals are available and allow pedigree-based approaches for building detailed linkage maps will be prime candidates for future research. Alternatively, obtaining a draft version of a genome with long continuous scaffolds is possible by massively parallel sequencing alone [39]. This opens up the unprecedented possibility of linking gene expression profiles to the physical, if not exact chromosomal location, so at least large scale scaffold information in basically any species.

## Conclusions

The lack of global dosage compensation in all ZZ/ZW systems that have so far been investigated reinstated much interest in studying the relationship between gene dosage and sex biased gene expression. So far, the study of dosage compensation has been limited to model organisms where a wealth of genetic tools is available. The advent of next generation sequencing techniques allows broadening the phylogenetic sample which will be central to our understanding how dosage compensation mechanisms evolve. We here exemplify this approach for a bird species, the European crow (*Corvus corone*), where previously no genomic resources were available.

In a first step, we established via qPCR that synteny to distantly related species with annotated genomes (zebra finch and chicken) is high. This allowed us placing crow contigs on the correct chromosome on the basis of orthology relationships. We then generated 454 transcriptome data for eight male and female individuals from which we derived a measure of digital gene expression (RNA-seq). Our data confirms the global lack of dosage compensation on the Z chromosome. In contrast to striking differences between microarray- and RNA-seq-based X-to-autosomal-expression values recently observed by Xiong et al. 2010 [22], our estimates of male-to-female expression difference on the Z that compare well to previous microarray-based estimates in birds and lepidopterae. The data further indicates that female up-regulation of Z-linked gene expression is responsible for partial compensation and establishes a link between the level of expression and sex-bias of a gene.

A potential limitation of our data set was the low read coverage, which introduced additional sampling variance and reduced statistical power. However, as technology progresses, this will be countered with an increased amount of sequencing data. Higher coverage data will also allow separating the effects of individual genes and relate them, for instance, to gene ontology terms. A more fundamental limitation, however, is given by the lack of gene order information that can only be inferred from other species with considerable uncertainty. Gene order information is important to address if localized mechanism of dosage compensation exist as has been suggested for the MHM locus in chicken. This challenge can be met by focusing on species with high quality linkage maps or by *de novo *assembly of a large number of reads into long scaffolds. The latter option now seems to be within reach even for individual labs [39]. Data on more species will eventually allow comparative analyses on the evolution of sex-biased gene expression. An indication from this study that the degree of compensation is partly conserved for over 100 million years of avian evolution makes this exciting prospect.

## Methods

### Taxonomic considerations

The Eurasian crow (*Corvus corone*) has two distinct colour morphs which are generally referred to as semi-species [40]: the all black carrion crow *(Corvus [corone] corone*) and the gray-coated and gray-bellied hooded crow (*Corvus [corone] cornix*). Phylogenies based on mtDNA [41] and several hundred nuclear markers (JBW Wolf, unpublished data), as well as population genetic studies based on RFLPs [42], microsatellites [43] and SNP data [44] fail to separate the two morphs. As dosage compensation is expected to be a feature shared not only on the species level, but potentially for birds and maybe even ZW systems in general, it is warranted to treat carrion and hooded crows as one species for the purpose of this study. We still conducted separate analysis for the morphs which qualitatively yielded the same results.

### Transcriptome sequencing

Wolf *et al*. 2010 [44] pyrosequenced barcoded cDNA samples from brain of 11 wild caught sub-adult crows (~2 years old) on a Roche 454 FLX sequencer. The resulting 856 675 raw reads from all individuals were assembled *de novo *into 19 552 contigs from which 7637 orthologues could be identified with zebra finch and chicken. For each of these genes individual expression levels were estimated as reads per million base pairs per kilo base exon (RPKM). For details of sampling regime, laboratory procedures and data processing see Wolf *et al*. 2010 [44]. Note that the individuals have been sampled in the wild and differences in age and environmental condition is expected to add variance to the expression data set. All results are therefore conservative and are likely to reflect rather strong effect sizes.

### Verification of Z-linkage with qPCR

In crows, inferences on chromosomal locations hinge on the plausible assumption of conserved synteny with other bird species [45]. To verify Z-linkage, we picked a subset of 21 crow genes for which orthology could be established with unique genes in zebra finch (Bio Mart, ENSEMBL 57). For each gene we aligned the coding sequence of zebra finch with all orthologous crow contigs mapping to the gene using CODONCODE ALIGNER VERSION 3.0.1. (CodonCode Corporation) and designed one to a maximum of six exonic primers with predicted product lengths of 50-170 bp using the software PRIMER3 [46] (Table 1 in Additional file 1).

In order to identify an appropriate control gene to which the signals from the putative Z-genes could be normalized, we compared amplification efficiency and variability of 12 putative autosomal genes selected on the basis of orthology to zebra finch and chicken. Two putative autosomal genes, ENSTGUG00000002932 and ENSTGUG00000013338 that showed similar amplification efficiency in both populations of samples, average m:f ratio of 1 and smallest Ct variability across samples were selected as normalizing genes. Results reported in Table 1 were normalized to the signal of ENSTGUG00000002932, and they were subsequently confirmed with ENSTGUG00000013338 (data not shown).

Amplification efficiency was assayed for 12 primer pairs amplifying 6 putative autosomal control genes and for 11 primer pairs amplifying 5 putative Z-linked genes by amplification of 4-log10 serial dilution of the template DNA (100 ng-100 pg). All samples were run in triplicates. For testing of the putative autosomal genes, we used a mix of template DNA from eight samples; for the putative Z-linked genes, we tested 4 samples, 2 from each population and sex. Primers used in these assays and amplification efficiencies are reported in Additional file 1 (Table 2 and 3, respectively).

qPCR reactions were run in triplicates on a total of eight individuals from both carrion and hooded crow populations (4 males and 4 females) with 5 or 10 ng DNA as a template using the FastSYBR protocol and chemistry (Applied Biosystems) on an ABI 7900HT machine (Applied Biosystems). Data was analyzed with the ΔCt method. The investigator was blind to the sexes of the animals tested.

The qPCR approach enabled us to measure the concentration of the putative Z linked genes relative to a putative autosomal reference gene. Deviations from the predicted mean ratio of 1 for males (ZZ), and 0.5 for females (ZW) are expected due to differences in the amplification efficiency of Z-linked primers and the autosomal control. More important than the absolute values for inference of Z-linkage is the ratio of mean male to mean female concentration: E (ZZ_conc_/ZW_conc_.) = 2. We used independent t-tests for each primer to assess the statistical significance of the difference.

### Dosage compensation analysis

#### a) Individuals and gene set

In our sample of the same eight barcoded individuals used in the qPCR analysis (two adult specimens from each sex and taxon) we find a total of 7564 transcripts for which orthology with chicken or zebra finch can be established. While the longer reads of the 454 technology are suitable for de novo transcriptome assembly, average per gene coverage for this data set is relatively low (6.3 reads per individual and gene). To reduce sampling variance for estimates of mean sex-specific gene expression we therefore restricted the analyses to genes that were expressed at above mean expression levels in at least two males and females and where log2(m:f ratios) did not exceed ±4. This results in a core set of 3556 moderately to highly expressed genes (11.5 reads per individual and gene). Further reduction of the gene set (i.e. all 1301 genes expressed in all eight individuals) would improve the estimates, but introduces a bias against Z-linked genes, as lowly expressed Z linked genes are often not expressed in females (due to a lack of dosage compensation). In the final set of 3556 genes, no such bias can be observed and - apart from a slight over-representation of genes on chromosome 2, 26 and 27 - the fraction of genes included for autosomes and the Z chromosome follows the expectation from zebra finch (Table 4 in Additional file 1). We therefore consider the 3556 gene set used as a good compromise trading-off equal genomic representation and precision in expression estimates.

#### b) GC content and RNA-seq

PCR amplification during next generation sequencing library preparation is not equally efficient across all genomic regions and depends on sequencing context. A relationship between GC content and RNA-seq estimates of gene expression is therefore expected and has been reported elsewhere [39]. To estimate the effect of GC content on expression we took GC values from zebra finch as a proxy for the GC content of a crow gene, since contigs are often only gene stubs. This assumption seems warranted as GC content is highly correlated even between chicken and zebra finch (r = 0.88, p > 0.001) that are twice as divergent. We find a significant relationship between GC content and male gene expression (F_3212 _= 254.5, p < 0.001), but no relationship with female expression (F_3212 _= 0.40 p > 0.05). This results in a significant relationship between GC content and the m:f ratio of gene expression (F_3212 _= 755.4 p < 0.001). This discrepancy is surprising and may possibly be explained by slight differences in coverage between males (11 reads per gene) and females (12 reads per gene) and the relative impact of GC bias in regions of higher and lower coverage.

GC content varies strongly between micro- and macro-chromosomes in birds and, unless controlled for, the observed GC bias in expression ratios leads to an underestimation of m:f ratios on large chromosomes (including Z) and overestimation of m:f ratios on small micro-chromosomes. As there is no indication for a biologically real difference in m:f ratios between chromosomes [11,12], the influence of GC was statistically removed. Analyses without controlling for GC qualitatively yield the same results though the m:f ratios of the relative GC-poor Z chromosome are lower.

#### c) Statistical analysis

Statistical evidence for decreased overall efficiency in dosage compensation was established as follows. First, we took the mean expression value for each sex and gene and calculated the m:f ratio. In the case of complete dosage compensation and equal overall expression in males and females this ratio is expected to be 1 across all autosomes and the Z chromosome alike. In the absence of any dosage compensation the ratio should be 1 for autosomes and 2 for genes on the Z chromosome. Data were normalized by log2 transformation which centres the expected values around 0 (log_2_(1) = 0) and 1 (log_2_(2) = 1). Deviations from the 0 baseline were tested in a linear regression framework. M:f expression ratios were regressed against chromosome identity as the categorical variable. By dropping the intercept an F-test tests the null hypothesis that all means are equal to 0 (no dosage compensation) and confidence intervals (Bonferroni corrected for the number of chromosomes) of the parameter estimates can be used to assess which of the chromosome deviates from 0. To account for samples size differences across chromosomes, the regression was weighted by the number of genes.

The Gaussian kernel density function shown in Figure 1 uses the algorithm implemented in the R density () function. The shape of the curve is robust to modifications of the default parameters even when different smoothing kernels were used.

### Cross-species comparison of m:f ratios

To prepare the data for the correlations across avian species (Table 2), we started with the chicken Unigene IDs reported in Tomaszycki et al. 2009 [30] for zebra finch (7299 genes) and obtained 1:1 orthologues between zebra finch and chicken Ensembl gene IDs (BioMart ENSEMBL Genes 59). In cases where multiple Unigene IDs corresponded to single Ensembl gene IDs, we took the average m:f ratios for a given Ensembl IDs and obtained a list of 3042 zebra finch - chicken 1:1 orthologues for which we had m:f ratios in zebra finch. We then assigned chicken m:f ratios from Mank et al. [13] (590 genes, unpublished) and crow m:f ratios (3556 genes) to this set, arriving at core set of 41 1:1:1 orthologues between chicken, zebra finch and crow.

## Authors' contributions

JW conceived of the study and conducted the dosage compensation analyses. JB was responsible for the design, execution and analysis of the qPCR part of the study. Both authors worked in the lab, analyzed the data and wrote the manuscript.

## Supplementary Material

Additional file 1 **Supplementary information**. The supplementary information contains one Figure on m:f ratios across orthologous chromosomal positions of zebra finch and chicken (Figure 1), additional information on the qPCR methodology (Table 1, 2-3) and sample sizes of genes used in the dosage compensation analysis (Table 4). Click here for file

## References

[B1] Mank JE Avise JC Evolutionary Diversity and Turn-Over of Sex Determination in Teleost Fishes Sex Dev 2009 3 2-3 60 67 10.1159/000223071 19684451

[B2] Sandra GE Norma MM Sexual determination and differentiation in teleost fish Rev Fish Biol Fish 2010 20 1 101 121 10.1007/s11160-009-9123-4

[B3] Ayling LJ Griffin DK The evolution of sex chromosomes Cytogenet Genome Res 2002 99 1-4 125 140 10.1159/000071584 12900555

[B4] Forstmeier W Ellegren H Trisomy and triploidy are sources of embryo mortality in the zebra finch Proc R Soc Lond B 2010 277 1694 2655 2660 10.1098/rspb.2010.0394 PMC2982043 20444723

[B5] Birchler JA Riddle NC Auger DL Veitia RA Dosage balance in gene regulation: biological implications Trends Genet 2005 21 219 226 10.1016/j.tig.2005.02.010 15797617

[B6] Batada NN Hurst LD Evolution of chromosome organization driven by selection for reduced gene expression noise Nature Genet 2007 39 8 945 949 10.1038/ng2071 17660811

[B7] Vicoso B Bachtrog D Progress and prospects toward our understanding of the evolution of dosage compensation Chromosome Res 2009 17 5 585 602 10.1007/s10577-009-9053-y 19626444 PMC2758192

[B8] Arnold AP Itoh Y Melamed E A bird's-eye view of sex chromosome dosage compensation Ann Rev Genom Hum G 2008 9 109 127 10.1146/annurev.genom.9.081307.164220 18489256

[B9] Mank JE The W, X, Y and Z of sex-chromosome dosage compensation Trends Genet 2009 25 5 226 233 10.1016/j.tig.2009.03.005 19359064 PMC2923031

[B10] Naurin S Hansson B Bensch S Hasselquist D Why does dosage compensation differ between XY and ZW taxa? Trends Genet 2010 26 1 15 20 10.1016/j.tig.2009.11.006 19963300

[B11] Ellegren H Hultin-Rosenberg L Brunstrom B Dencker L Kultima K Scholz B Faced with inequality: chicken do not have a general dosage compensation of sex-linked genes Bmc Biology 2007 5 10.1186/1741-7007-5-40 17883843 PMC2099419

[B12] Itoh Y Melamed E Yang X Kampf K Wang S Yehya N Van Nas A Replogle K Band M Clayton D Dosage compensation is less effective in birds than in mammals J Biol 2007 6 1 2 10.1186/jbiol53 17352797 PMC2373894

[B13] Mank JE Ellegren H All dosage compensation is local: Gene-by-gene regulation of sex-biased expression on the chicken Z chromosome Heredity 2009 102 3 312 320 10.1038/hdy.2008.116 18985062

[B14] Itoh Y Replogle K Kim Y-H Wade J Clayton DF Arnold AP Sex bias and dosage compensation in the zebra finch versus chicken genomes: General and specialized patterns among birds Genome Res 2010 20 4 512 518 10.1101/gr.102343.109 20357053 PMC2847754

[B15] Birchler JA Fernandez HR Kavi HH Commonalities in compensation BioEssays 2006 28 565 568 10.1002/bies.20408 16700062

[B16] Melamed E Arnold AP Regional differences in dosage compensation on the chicken Z chromosome Genome Biol 2007 8 9 10.1186/gb-2007-8-9-r202 17900367 PMC2375040

[B17] Zha XF Xia QY Duan J Wang CY He NJ Xiang ZH Dosage analysis of Z chromosome genes using microarray in silkworm, Bombyx mori Insect Biochem Molec 2009 39 5-6 315 321 10.1016/j.ibmb.2008.12.003 19150406

[B18] McQueen HA Clinton M Avian sex chromosomes: dosage compensation matters Chromosome Res 2009 17 5 687 697 10.1007/s10577-009-9056-8 19802708

[B19] Zhang Y Malone JH Powell SK Periwal V Spana E MacAlpine DM Oliver B Expression in Aneuploid Drosophila S2 Cells PLoS Biol 2010 8 2 10.1371/journal.pbio.1000320 PMC2826376 20186269

[B20] McAnally AA Yampolsky LY Widespread Transcriptional Autosomal Dosage Compensation in Drosophila Correlates with Gene Expression Level Genome Biol Evol 2010 2010 0 44 52 10.1093/gbe/evp054 PMC2839349 20333221

[B21] Ruiz MF Esteban MR Donoro C Goday C Sanchez L Evolution of dosage compensation in diptera: The gene maleless implements dosage compensation in Drosophila (Brachycera suborder) but its homolog in sciara (Nematocera suborder) appears to play no role in dosage compensation Genetics 2000 156 4 1853 1865 11102379 10.1093/genetics/156.4.1853 PMC1461397

[B22] Xiong YY Chen XS Chen ZD Wang XZ Shi SH Wang XQ Zhang JZ He XL RNA sequencing shows no dosage compensation of the active X-chromosome Nature Genet 2010 42 12 1043 U1029 10.1038/ng.711 21102464

[B23] Tautz D Ellegren H Weigel D Next Generation Molecular Ecology Mol Ecol 2010 19 Suppl. 1 1 3 10.1111/j.1365-294X.2009.04489.x 20331765

[B24] Wang Z Gerstein M Snyder M RNA-Seq: a revolutionary tool for transcriptomics Nature Rev Genet 2009 10 1 57 63 10.1038/nrg2484 19015660 PMC2949280

[B25] Ellegren H Evolutionary stasis: the stable chromosomes of birds Trends Ecol Evol 2010 25 5 283 291 10.1016/j.tree.2009.12.004 20363047

[B26] Innocenti P Morrow EH The Sexually Antagonistic Genes of *Drosophila melanogaster* PLoS Biol 2010 8 3 e1000335 10.1371/journal.pbio.1000335 20305719 PMC2838750

[B27] Mane S Evans C Cooper K Crasta O Folkerts O Hutchison S Harkins T Thierry-Mieg D Thierry-Mieg J Jensen R Transcriptome sequencing of the Microarray Quality Control (MAQC) RNA reference samples using next generation sequencing BMC Genomics 2009 10 1 264 10.1186/1471-2164-10-264 19523228 PMC2707382

[B28] Oshlack A Wakefield M Transcript length bias in RNA-seq data confounds systems biology Biol Direct 2009 4 1 14 10.1186/1745-6150-4-14 19371405 PMC2678084

[B29] Altug-Teber O Bonin M Walter M Mau-Holzmann UA Dufke A Stappert H Tekesin I Heilbronner H Nieselt K Riess O Specific transcriptional changes in human fetuses with autosomal trisomies Cytogenet Genome Res 2007 119 3-4 171 184 10.1159/000112058 18253026

[B30] Birchler JA Bhadra U Bhadra MP Auger DL Dosage-dependent gene regulation in multicellular eukaryotes: implications for dosage compensation, aneuploid syndromes, and quantitative traits Dev Biol 2001 234 275 288 10.1006/dbio.2001.0262 11396999

[B31] Tomaszycki ML Peabody C Replogle K Clayton DF Tempelman RJ Wade J Sexual differentiation of the zebra finch song system: potential roles for sex chromosome genes BMC Neurosci 2009 10 10.1186/1471-2202-10-24 19309515 PMC2664819

[B32] Griffin DK Robertson LBW Tempest HG Skinner BM The evolution of the avian genome as revealed by comparative molecular cytogenetics Cytogenet Genome Res 2007 117 1-4 64 77 10.1159/000103166 17675846

[B33] Backstrom N Brandstrom M Gustafsson L Qvarnstrom A Cheng H Ellegren H Genetic Mapping in a Natural Population of Collared Flycatchers (*Ficedula albicollis*): Conserved Synteny but Gene Order Rearrangements on the Avian Z Chromosome Genetics 2006 174 377 386 10.1534/genetics.106.058917 16783008 PMC1569790

[B34] Dawson DA Burke T Hansson B Pandhal J Hale MC Hinten GN Slate J A predicted microsatellite map of the passerine genome based on chicken-passerine sequence similarity Mol Ecol 2006 15 5 1299 1320 10.1111/j.1365-294X.2006.02803.x 16626455

[B35] Hansson B Ljungqvist M Dawson DA Mueller JC Olano-Marin J Ellegren H Nilsson JA Avian genome evolution: insights from a linkage map of the blue tit (Cyanistes caeruleus) Heredity 2010 104 1 67 78 10.1038/hdy.2009.107 19707235

[B36] Jaari S Li MH Merila J A first-generation microsatellite-based genetic linkage map of the Siberian jay (Perisoreus infaustus): insights into avian genome evolution BMC Genomics 2009 10 1 10.1186/1471-2164-10-1 19121221 PMC2671524

[B37] Backstrom N Forstmeier W Schielzeth H Mellenius H Nam K Bolund E Webster MT Ost T Schneider M Kempenaers B The recombination landscape of the zebra finch Taeniopygia guttata genome Genome Res 2010 20 4 485 495 10.1101/gr.101410.109 20357052 PMC2847751

[B38] Nanda I Schlegelmilch K Haaf T Schartl M Schmid M Synteny conservation of the Z chromosome in 14 avian species (11 families) supports a role for Z dosage in avian sex determination Cytogenet Genome Res 2008 122 2 150 156 10.1159/000163092 19096210

[B39] Li RQ Fan W Tian G Zhu HM He L Cai J Huang QF Cai QL Li B Bai YQ The sequence and de novo assembly of the giant panda genome Nature 2010 463 7279 311 317 10.1038/nature08696 20010809 PMC3951497

[B40] Parkin DT Collinson M Helbig AJ Knox AG Sangster G The taxonomic status of Carrion and Hooded Crows British Birds 2003 96 274 290

[B41] Haring E Gamauf A Kryukov AP Phylogeographic patterns in widespread corvid birds Mol Phylogenet Evol 2007 45 3 840 862 17920300 10.1016/j.ympev.2007.06.016

[B42] Kryukov AP Uphyrkina OV Chelomina GN Analysis Of Crow Genomes (Corvidae, Passeriformes) From The Zone Of Overlapping Areas Of Habitation And Hybridization Genetika 1992 28 6 136 140

[B43] Haas F Pointer MA Saino N Brodin A Mundy NI Hansson B An analysis of population genetic differentiation and genotype-phenotype association across the hybrid zone of carrion and hooded crows using microsatellites and MC1R Mol Ecol 2009 18 2 294 305 10.1111/j.1365-294X.2008.04017.x 19076276

[B44] Wolf JBW Bayer T Haubold B Schilhabel M Rosenstiel P Tautz D Nucleotide divergence versus gene expression differentiation: comparative transcriptome sequencing in natural isolates from the carrion crow and its hybrid zone with the hooded crow Mol Ecol 2010 19 162 175 10.1111/j.1365-294X.2009.04471.x 20331778

[B45] Backstrom N Karaiskou N Leder EH Gustafsson L Primmer CR Qvarnstrom A Ellegren H A gene-based genetic linkage map of the collared flycatcher (Ficedula albicollis) reveals extensive synteny and gene-order conservation during 100 million years of avian evolution Genetics 2008 179 3 1479 1495 10.1534/genetics.108.088195 18562642 PMC2475748

[B46] Rozen S Skaletsky HJ Krawetz S, Misener S Primer3 on the WWW for general users and for biologist programmers Bioinformatics Methods and Protocols: Methods in Molecular Biology 2000 Totowa, NJ: Humana Press 365 386 10.1385/1-59259-192-2:365 10547847

