# Supplementary material for: General lack of global dosage compensation in ZZ/ZW systems? Broadening the perspective with RNA-seq
Source: BMC Genomics. 2011 Feb 1;12:91. doi: 10.1186/1471-2164-12-91 (PMC3040151; doi:10.1186/1471-2164-12-91)
Supplement: Additional file 1 — Supplementary information. The supplementary information contains one Figure on m:f ratios across orthologous chromosomal positions of zebra finch and chicken (Figure 1), additional information on the qPCR methodology (Table 1, 2-3) and sample sizes of genes used in the dosage compensation analysis (Table 4). [file 1471-2164-12-91-S1.PDF]

General lack of global dosage compensation in ZZ/ZW systems? Broadening the perspective with RNA-seq

Jochen B. W. Wolf, Jarosław Bryk

Supplementary information

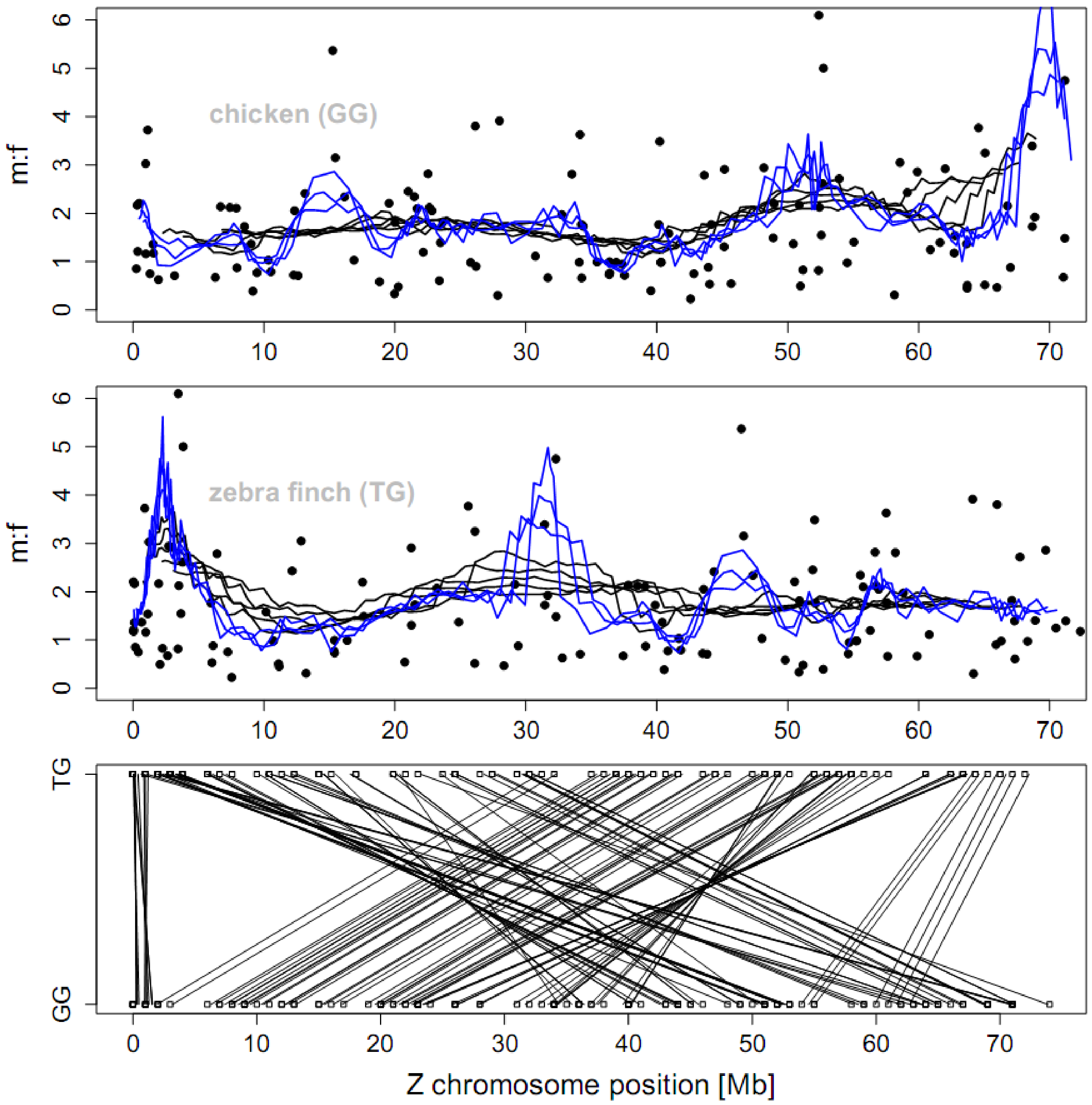

Supplementary Figure 1: Male to female ratios (m:f) of expression as a function of chromosomal location as inferred from orthology with the chicken Z chromosome (upper panel) and the zebra finch Z chromosome (lower panel). Points represent any of 127 genes with orthology relationships to both zebra finch and chicken. Lines represent sliding window averages averaging across 15, 20, 25, 30, 35 neighbouring genes (black lines) and over 5,7,9 genes (blue lines). The lower panel indicates rearrangements between zebra finch (ZF) and chicken (GG) for the genes.

Supplementary Table 1: Primer sequences and amplicon lenghts used in quantitative PCR analyses for the putative Z-linked genes.

Gene ID	Gene region	Internal primer code	Forward primer	Reverse primer	Amplicon length [bp]
ENSGALG00000012606	exon 4	Z1	CTTCAGATTGCATTTCTCTGC	CACCAGCACTCTTAGCAACC	81
	3' UTR	Z2	TGAAAACCtGGAGACAGACC	CGGGTCAGTaACAGAATGC	106
ENSTGUG00000000043	exon 3	Z3	CTACATCTACCCCGTGATCG	ATCCTCCTGGTCTCTTTTGG	145
	exon 4	Z37	GGTGAAGAACTGTGGACTGC	GCATAACTGCTTTAGCTCATCC	105
	exon 4	Z38	AATGGAACCACTGCAAAGAG	GAAAGTCAAAGGAATATCAGCAG	122
ENSTGUG00000000468	exon 4	Z4	CAATGATTGGTAAAGCTGTGG	CCTcCACTCATCTTTTGAGC	65
	3' UTR	Z5	tCTGGCTTTTCCACATAAGG	CACAAAAGGCAACAACAGG	66
ENSTGUG00000000903	3' UTR	Z6	TGCCACAAGAAAATGAAGC	CATTCCAGAAACACATCAAGG	133
	exon 26	Z7	AAGATGATGGCACAGATTCC	AATGGAGAACTCAAAGGtTGG	94
	3' UTR	Z8	TGTGGCTTTCTTATTCAGTGC	GATTGCTCCCACTATAACTGC	112
ENSTGUG00000001006	exon 1	Z9	ATGCTGCCCTTTTGTAATCC	CCCTGCTCACTTTCTGTCC	66
	exon 4	Z10	GAATCGCAAGAAAAGTGAGG	TGGTGAAATGTTGTCCATAGC	71
ENSTGUG00000001019	exon 4	Z11	CGTGCAGTACAGAGGTAGGG	TTGGAAACTGAGGTAAACACG	74
	exon 5	Z12	GGAGGCAAGAAAGGTAAAGG	AGCAGGCAGTTCTACTCAGC	161
ENSTGUG00000001401	3' UTR	Z13	CTGCCTCTTTTAACACTACTTGC	AATCAACAATCTGCACTCAGG	102
	3' UTR	Z14	AGACTTGTGTCATTCCAGAGC	TGGTTTATTGGTgAAaCTGG	170
ENSTGUG00000001582	exon 5	Z15	AAGACACGTAGGAGAGTTGG	TGTCCACAGCCTTAATCC	94
ENSTGUG00000001587	3' UTR	Z16	CATGGTTCTGGTGCTAGAGG	TTTATAGGCTCGCAGATAGAGC	134
	3' UTR	Z17	TGTGGAATGTTTCATGTTTAGC	AGTTGAAGGGCTCATATTTCC	73
ENSTGUG00000001918	3' UTR	Z18	TGGAGAACTTGGAGATGTAAGG	TtGTTTTATCTTCTTGCTTTGC	155
	3' UTR	Z19	GAGTCACAAACAATGGAAGG	AATTAACTCCTCCAGCTCTGC	155
ENSTGUG00000002186	exon 2	Z20	GGAGTGCCAAGGCTAAACG	CCTCATGCGACCAGTACC	50
	exon 2	Z21	AGGGTACATCGTCGTTTGG	GCAGATGGTATGCCTTGG	81
ENSTGUG00000002229	3' UTR	Z22	GAACGGAGCTAGTGTGAAGG	GGGGAGAAGCATTATATCTGG	118
	exon 5	Z23	GGCCGTCACtTTATTCtGG	CTTCACAAGAGCAAAATCTCC	54
ENSTGUG00000003373	exon 5	Z24	GTGTGGAATTTGACTTTGTACC	TTTGGACAGGAAGTTTGAGG	77
ENSTGUG00000003968	exon 3	Z25	ATTAGCACGGGGTCTATTCC	CACCTTGCAGTTAGAGAAAGC	108
ENSTGUG00000004334	exon 4	Z26	TAAGAAACCCAGAACGAAGG	ACAGCTTGGCGTACTCTGC	140
ENSTGUG00000006083	exon 5	Z27	AAGGCTACATTTGTGGTTGG	GAACACATTCCTCAATAGAACG	115
ENSTGUG00000006814	exon 2	Z28	AACCTCCCATTCTCTGTGG	TGTCCTCTTCATTTCTTCAGG	125
ENSTGUG00000006923	exon 4	Z29	CTCCAGAAGACAGGTGATCC	TGTGCTGTGTGGTGATGG	159
	exon 12	Z30	ACCAACCTTAGTGCAGACG	GGTAGAGCTGGGATGACtGG	101
	exon 13	Z32	ACTCACATTGAAGCGGAATG	CGACATCACACACCAAGTTC	79
ENSTGUG00000017495	exon 17	Z33	CTCATGGAGGATACGGTTTG	GCGTGGATAGAGACAGACATC	89
	3' UTR	Z34	TGGTAACTGAAGCACACTGG	CAGTAGCACAAGGGAACAGG	106
	3' UTR	Z35	TTTTCTCTTGGGGTTTCTTG	TCCTTTGCTCATGTCTCTCC	101
	exon 9	Z36	GGGCTGACAATCTGCTTAATG	ATCATGTGCCtGTTGTTGAG	64
ENSTGUG00000017541	3' UTR	Z31	GACGCTGTGTAATGACTTGC	TGGTGAACACGAAAGTTAAGC	136

Supplementary Table 2: Primer sequences for putative autosomal genes used to select normalizing control genes. Primers selected and used in further analyses are depicted in **bold**. Note that results reported in Table 1 were obtained using ENSTGUG00000002932 as a control gene, and subsequently confirmed using ENSTGUG00000013338 (data not shown).

Gene ID	Gene region	Internal primer code	Forward primer	Reverse primer	Amplicon length (bp)
ENSTGUG00000002022	exon 12	A1	TCCAGATCCTGCACATCC	GCTCGAACCAAACCTTCC	130
	exon 8	A2	AGGGCATCCTCCTGTACG	CTCTGCATCAGCAAACAGC	170
ENSTGUG00000002932	exon 8	A3	ATTTTGAAAGAGCAGCTTCG	TTATCCCGCACATTtACTGC	107
	**exon 3**	**A4**	**GAAATCCGTGGAGCAAGC**	**CACCACTAACATATCAGAGTTTCC**	**102**
ENSTGUG00000011788	exon 4	A5	TTCCCATGACACTTACTTCTACC	TGTCCAGAAAGGATAAACACG	79
	exon 13	A6	GGTGCTGTGAATGAATTTGG	GACACAACCTCCTGAAATGC	100
ENSTGUG00000012606	exon 5	A7	AAAATGAAGACCCAAGAGAGG	CTCAGCTTCCTCCTGATCC	105
	exon 6	A8	AGGATGTGAGAAGGTCATGG	CTTCTTTTCTTCAGGCAtGG	89
ENSTGUG00000013112	3'-UTR	A9	TCACAaGTTCACAaCCAaGC	tgtgaatcacgtttcaatgC	106
	exon 5	A10	GaCGCTCAGAGGaatGAGG	ACATTCCGCAAAAGATTAGG	104
ENSTGUG00000013338	exon 11	A11	CGTGTTGTGGACTTGATGG	TAAGCGGTGGTGAACAAGG	123
	**exon 7**	**A12**	**CACACAGAAGACAGTGGATGG**	**ACTTTTCCCACAGCCTTAGC**	**111**

Supplementary Table 3: Amplification efficiencies assayed for 12 assays amplifying 6 putative autosomal genes and 11 assays amplifying 5 putative Z-linked genes. Slope, R^2 and efficiency values were calculated according to [1]. For the purpose of this study, the efficiency values needed to be similar across sexes and not necessarily between assays amplifying putative autosomal and Z-linked genes. Slight differences among samples and among assays amplifying putative autosomal and Z-linked genes affected the expected ratio of autosomal to Z-linked concentration in each sample. The ratio of mean male to mean female concentration: E ($ZZ_{conc.}$ / $ZW_{conc.}$) = 2, however, proved robust and consistent across assays.

Template DNA	Internal primer code	slope	R^2	Efficiency $E=10^{(-1/slope)}-1$
Carrion (mix)	A1	-3,3053	0,9999	101%
Hooded (mix)		-3,4536	1	95%
Carrion (mix)	A2	-3,8608	0,9948	82%
Hooded (mix)		-3,526	1	92%
Carrion (mix)	A3	-3,1671	0,9984	107%
Hooded (mix)		-3,2324	0,9991	104%
Carrion (mix)	A4	-3,2484	0,9999	103%
Hooded (mix)		-3,3388	0,9982	99%
Carrion (mix)	A5	-3,3848	0,9997	97%
Hooded (mix)		-3,5028	0,9996	93%
Carrion (mix)	A6	-3,469	0,9997	94%
Hooded (mix)		-3,4855	0,9993	94%
Carrion (mix)	A7	-1,8791	0,9068	241%
Hooded (mix)		-2,256	0,9388	177%
Carrion (mix)	A8	-2,9373	0,99	119%
Hooded (mix)		-2,7849	0,9956	129%
Carrion (mix)	A9	-3,7466	0,9982	85%
Hooded (mix)		-3,3928	0,9981	97%
Carrion (mix)	A10	-3,7429	0,9991	85%
Hooded (mix)		-3,5244	1	92%
Carrion (mix)	A11	-3,1521	0,9992	108%
Hooded (mix)		-3,2946	0,9999	101%
Carrion (mix)	A12	-3,1118	0,999	110%
Hooded (mix)		-3,1584	0,9995	107%
Carrion female 1	Z4	-3,2289	0,9995	104%
Carrion male 1		-4,0792	0,9794	76%
Hooded female 1		-3,2365	0,9998	104%
Hooded male 1		-3,2891	0,9968	101%
Carrion female 1	Z10	-2,9370	0,9716	119%
Carrion male 1		-3,6321	0,9917	89%
Hooded female 1		-3,4455	0,9982	95%
Hooded male 1		-3,4075	0,9999	97%
Carrion female 1	Z12	-2,6498	0,9462	138%
Carrion male 1		-3,3242	0,9962	100%
Hooded female 1		-3,5693	0,9986	91%
Hooded male 1		-3,4689	0,9985	94%
Carrion female 1	Z32	-3,1796	0,9997	106%
Carrion male 1		-3,5311	0,9941	92%
Hooded female 1		-3,2507	0,9999	103%
Hooded male 1		-3,1934	1	106%
Carrion female 1	Z33	-3,3835	0,9985	97%
Carrion male 1		-3,354	0,9975	99%
Hooded female 1		-3,4326	0,9986	96%
Hooded male 1		-3,3152	0,9993	100%
Carrion female 1	Z34	-2,9448	0,9996	119%
Carrion male 1		-2,9132	0,9995	120%
Hooded female 1		-2,8738	0,9985	123%

Hooded male 1		-2,8993	0,999	121%
Carrion female 1	Z35	-3,6443	0,9984	88%
Carrion male 1		-3,3864	0,9994	97%
Hooded female 1		-3,2012	0,9986	105%
Hooded male 1		-3,2912	0,9997	101%
Carrion female 1	Z36	-3,4776	0,9996	94%
Carrion male 1		-3,3136	0,9986	100%
Hooded female 1		-3,1717	0,9985	107%
Hooded male 1		-3,1909	0,9974	106%
Carrion female 1	Z37	-2,8855	0,9888	122%
Carrion male 1		-3,1814	0,9957	106%
Hooded female 1		-3,4597	0,9996	95%
Hooded male 1		-3,2325	0,9992	104%
Carrion female 1	Z38	-3,0893	0,9797	111%
Carrion male 1		-3,176	0,9993	106%
Hooded female 1		-3,1242	0,9971	109%
Hooded male 1		-3,1744	0,9989	107%

Supplementary Table 4: Number of genes included in the analysis in comparison to the total number of genes from zebra finch (Biomart ENSEMBL59, x_random included in x). χ^2 residuals indicate on which chromosomes the observed number of genes significantly differs from the expectation. Asterisks denote the standard Type I error probabilities.

chromosome	zebra finch	crow observed	crow expected	residuals
**1**	1179	298	281	1.00
**1A**	930	226	220	0.39
**1B**	37	6	8	-0.76
**2**	1394	390	340	2.73***
**3**	1238	320	297	1.35
**4**	777	201	186	1.08
**4A**	364	82	85	-0.32
**5**	956	258	231	1.76
**6**	573	130	134	-0.34
**7**	538	123	126	-0.26
**8**	555	121	129	-0.68
**9**	471	105	110	-0.45
**10**	428	97	100	-0.30
**11**	379	74	86	-1.32
**12**	354	83	83	-0.02
**13**	364	108	90	1.91
**14**	413	90	96	-0.59
**15**	373	98	90	0.88
**17**	321	60	73	-1.47
**18**	315	57	71	-1.64
**19**	339	79	80	-0.07
**20**	346	89	83	0.68
**21**	248	49	57	-1.01
**22**	126	30	30	0.05
**23**	232	48	53	-0.73
**24**	197	32	44	-1.76
**25**	126	18	27	-1.80
**26**	239	37	53	-2.15***
**27**	267	42	59	-2.20***
**28**	244	49	56	-0.91
**Z**	793	156	181	-1.84
